# The Voluntary Hospitals Committee

**Published:** 1921-06-11

**Authors:** 


### The Voluntary Hospitals Committee.

SUMMARY OF REPORT AND RECOMMENDATIONS.

1. The present financial difficulties of the hospitals are due to the war. The receipts from voluntary subscriptions, donations, and legacies have not fallen off; on the contrary, they have risen by 67 per cent. since 1913. But during the war the cost of provisions, drugs, dressings, fuel, and labour has increased to such an extent that the expenditure of the hospitals has increased by 138 per cent. since 1913. The aggregate deficits of all the voluntary hospitals of Great Britain for the present year are estimated at £1,000,000.

2. The voluntary system is therefore temporarily in jeopardy. The Committee ask: "Is the voluntary system worth saving?" and their conclusion is, "We are convinced that it is." Apart from the immense sum of voluntary subscriptions and donations (estimated at £3,000,000 a year), the voluntary system secures gratuitous and skilled service, both lay and medical, of incalculable advantage. It preserves freedom in medicine, and is of inestimable value to the welfare of the sick, the training of the medical profession, and the progress of medical research. The breakdown of the voluntary system would imperil these services and impose a constantly growing charge upon public funds.

3. The Committee are of opinion that if the voluntary hospitals are to be saved immediate assistance must be given by the State, such assistance to be strictly limited to a definite period of two years, and to be given only to hospitals which show that they are taking all possible steps to re-establish their financial position. A grant so limited and conditioned would re-establish the hospitals in the self-supporting position which they occupied before the war, and would not be inconsistent with the maintenance of the voluntary system.

4. The Committee draw attention to a number of steps which managers of voluntary hospitals can themselves take to improve the financial position of the hospitals under their charge—*e.g.*, review of expenditure, particularly in the light of comparative costs, which could be ascertained by the general adoption of a uniform system of accounts; co-operative buying of drugs, apparatus, stores, and some kinds of provisions; removal of convalescent cases to auxiliary hospitals; co-ordination of appeals for subscriptions; further development of new sources of income, such as subscriptions from wage-earners and employers, and other contributory schemes, examples of which are quoted in the Report; contributions from Approved Societies.

Recommendations.

5. To give effect to the temporary State assistance the Committee make the following recommendations:—

(*a*) That a Hospital Commission should be set up by the Minister of Health.

(*b*) That Voluntary Hospitals Committees be formed in each county or county borough; the King Edward's Hospital Fund to act for London.

(*c*) That Parliament be asked to sanction a temporary grant of £1,000,000 for 1921, to be administered by the Hospitals Commission; grants to be made normally on the recommendations of the local Voluntary Hospitals Committees.

(*d*) That a further grant may be required in 1922, but no Government assistance to be promised beyond that year.

(*e*) That the Hospitals Commission during the two years may recommend grants (not exceeding £250,000 in 1921-22) for extension and improvement of hospitals, subject to like contributions being made from private sources.

(*f*) That arrangements should be negotiated through the Voluntary Hospitals Committees for the utilisation of vacant beds in Poor-law infirmaries.

(*g*) That contributions by employers to hospital funds should be allowed as deductions from profits for income-tax purposes, and testamentary gifts to hospitals should be relieved of legacy and succession duty.

(*h*) That County Councils be empowered to contribute to the expenses of Voluntary Hospitals Committees.

(*i*) That, failing the provision in the National Health Insurance Acts of a "hospital benefit," the Courts be authorised to award to hospitals compensation under the Employers' Liability and Workmen's Compensation Acts.

(*j*) That local authorities be authorised to pay the cost of the treatment in hospitals of persons in their employ.

(*k*) That the payment out of technical education funds of grants for the training of nurses be considered.

(*l*) That provision be made for obtaining and tabulating returns of cases treated in hospitals.

(*m*) That where the payment to a hospital of a testamentary gift of residue is delayed for more than a year, the hospital be authorised to claim repayment of income tax.

(*n*) That legacy and succession duty on testamentary gifts to hospitals be remitted.

